# Clinicopathological Correlation of Kidney Allograft Biopsies According to the Banff 2022 Classification

**DOI:** 10.7759/cureus.114482

**Published:** 2026-08-13

**Authors:** Osmany Reyes Luaces, Gerardo Borroto Díaz

**Affiliations:** 1 Nephrology, Hospital Clínico Quirúrgico Hermanos Ameijeiras, Havana, CUB

**Keywords:** allograft function, banff 2022 classification, kidney allograft biopsy, kidney allograft rejection, kidney transplantation

## Abstract

Introduction

Kidney allograft biopsy remains the gold standard for the diagnosis of allograft dysfunction, providing essential information for identifying immune- and non-immune-mediated injuries. The Banff 2022 Classification represents the current international standard for the histopathological evaluation of kidney transplant biopsies. This study aimed to describe the clinicopathological distribution of kidney allograft biopsies according to the Banff 2022 Classification and to assess graft function 12 months after the index biopsy.

Methods

A retrospective descriptive study was conducted involving 119 kidney transplant recipients who underwent kidney allograft biopsy at Hospital Hermanos Ameijeiras, Havana, Cuba, between January 2006 and December 2018. Archived biopsy specimens were re-evaluated by an experienced renal pathologist according to the Banff 2022 Classification. Demographic characteristics, histopathological diagnoses, time from transplantation to biopsy, and graft function 12 months after the index biopsy were analyzed using descriptive statistics. Graft function was assessed by estimated glomerular filtration rate (eGFR) and proteinuria.

Results

Male recipients and patients younger than 60 years predominated in the study population. Non-rejection causes of allograft injury represented the largest diagnostic group, accounting for 52 (43.7%) biopsies, followed by T cell-mediated rejection (TCMR) in 37 (31.1%) and antibody-mediated rejection (ABMR) in 14 (11.8%). Histopathological diagnoses exhibited distinct temporal patterns after transplantation. During the first three months, acute cyclosporine nephrotoxicity was the most frequent diagnosis, followed by acute tubular necrosis (ATN) and acute TCMR, whereas after the first post-transplant year, chronic active TCMR was the most frequent diagnosis, followed by chronic active antibody-mediated rejection and chronic cyclosporine nephrotoxicity. At 12 months after the index biopsy, immune-mediated lesions generally showed lower mean eGFR values and more frequent proteinuria, whereas normal biopsies and non-immunological lesions, particularly ATN and acute cyclosporine nephrotoxicity, showed comparatively better graft function.

Conclusions

Kidney allograft biopsies demonstrated a broad spectrum of histopathological diagnoses, with non-rejection causes of allograft injury representing the most frequent findings. Histopathological diagnoses showed distinct temporal patterns according to the time from transplantation to biopsy, with acute cyclosporine nephrotoxicity predominating during the first three months after transplantation. At 12 months after the index biopsy, immune-mediated lesions generally showed lower eGFR and more frequent proteinuria, whereas normal biopsies and non-immune-mediated lesions showed comparatively better graft function.

## Introduction

Kidney transplantation is the treatment of choice for patients with end-stage kidney disease (ESKD), providing superior survival, improved quality of life, and greater cost-effectiveness than long-term dialysis. Advances in donor selection, organ preservation, surgical techniques, immunosuppressive therapy, and post-transplant care have significantly improved transplant outcomes. In 2024, a total of 173,727 solid organ transplants were reported worldwide, of which 110,467 (63.6%) were kidney transplants, making kidney transplantation the most frequently performed solid organ transplant worldwide [1].

Percutaneous kidney allograft biopsy remains the gold standard for evaluating allograft dysfunction. By providing direct histopathological evidence of tissue injury, it enables the accurate diagnosis of allograft rejection and other pathological processes affecting the transplanted kidney. In addition to establishing the diagnosis, biopsy findings provide important prognostic information and guide therapeutic decisions, making biopsy interpretation a cornerstone of kidney transplant management [2,3].

To standardize the interpretation of kidney allograft biopsies, the Banff Classification for Renal Allograft Pathology was introduced in 1991 as an international consensus-based classification system. Since then, it has evolved to incorporate advances in transplant pathology and immunology, refining the diagnosis of T cell-mediated rejection (TCMR) and antibody-mediated rejection (ABMR) while integrating molecular diagnostics. The Banff 2022 update further refined the assessment of microvascular inflammation/injury (MVI), clarified the diagnosis of C4d-negative ABMR, and strengthened the role of transcriptomics in transplant diagnostics [4,5].

Although the Banff Classification has standardized the interpretation of kidney allograft biopsies, histopathological findings should not be interpreted in isolation. Accurate interpretation requires integration of biopsy findings with the clinical presentation and laboratory data to determine the significance of pathological lesions and guide clinical management. Furthermore, the distribution of histopathological findings may vary among transplant centers because of differences in patient characteristics and clinical practices. Institutional studies can therefore provide valuable information regarding local patterns of allograft pathology and graft function [6].

The Banff Classification has been routinely used to interpret kidney allograft biopsies at our institution since 1997. However, no previous institutional study has described histopathological findings in relation to clinical parameters and subsequent graft function in our transplant population. Therefore, this study aimed to describe the clinicopathological findings of kidney allograft biopsies according to the Banff 2022 Classification and assess graft function 12 months after the index biopsy.

## Materials and methods

Study design

A retrospective, descriptive, single-center observational study was conducted at the Department of Nephrology, Hospital Clínico Quirúrgico Hermanos Ameijeiras, Havana, Cuba. The study aimed to describe the clinicopathological findings of kidney allograft biopsies retrospectively re-evaluated according to the Banff 2022 Classification and to assess graft function 12 months after the index biopsy.

Study population

The study included 119 unique kidney transplant recipients who underwent kidney allograft biopsy between January 2006 and December 2018 at Hospital Clínico Quirúrgico Hermanos Ameijeiras. Clinical, laboratory, and histopathological data were retrospectively obtained from the corresponding medical records and pathology archives. The inclusion and exclusion criteria for study selection are summarized in Table 1.

**Table 1:** Inclusion and exclusion criteria

Inclusion criteria	Exclusion criteria
Kidney allograft biopsy performed at Hospital Clínico Quirúrgico Hermanos Ameijeiras	Missing clinical or pathological data
Kidney transplantation performed at the same institution	Inadequate biopsy specimen for diagnosis
Histopathological specimen considered diagnostically adequate according to the Banff adequacy criteria	Graft survival of less than 12 months after the index biopsy
Availability of 12 month clinical follow-up after the index biopsy	

Kidney allograft biopsy procedure

All kidney allograft biopsies were performed using a percutaneous ultrasound-guided technique according to institutional protocols. Biopsies were performed either as protocol biopsies or for the evaluation of graft dysfunction. Biopsy specimens were processed in the Department of Pathology and evaluated by an experienced renal pathologist with expertise in kidney transplant pathology. Specimens were considered diagnostically adequate when they contained at least 10 glomeruli, representative tubulointerstitial tissue, and a minimum of two arteries, in accordance with the Banff adequacy criteria.

Routine histopathological evaluation included hematoxylin and eosin, periodic acid-Schiff, Jones methenamine silver, and Masson's trichrome staining. Immunofluorescence studies were performed using antibodies against IgG, IgA, IgM, C3, and C1q. C4d was assessed by immunohistochemistry on paraffin-embedded biopsy tissue. Immunohistochemical testing for viral pathogens, including SV40 and cytomegalovirus, was not available during the study period; therefore, cases with histopathological findings suggestive of viral-associated tubulointerstitial nephritis could not be definitively assigned to a specific viral etiology.

Histopathological evaluation

All kidney allograft biopsy specimens were retrospectively re-examined and classified according to the Banff 2022 Classification for Renal Allograft Pathology.

Biopsies were classified into six diagnostic categories: Category 1, normal biopsy or nonspecific changes; Category 2, ABMR and MVI; Category 3, suspicious (borderline) for acute TCMR; Category 4, TCMR; Category 5, interstitial fibrosis and tubular atrophy (IFTA); and Category 6, other changes not considered to be caused by rejection.

For Category 2 lesions, ABMR was diagnosed based on the required histopathological criteria and positive C4d staining. All cases classified as ABMR were C4d positive. Donor-specific antibody (DSA) testing and molecular diagnostics were not available during the study period; therefore, positive C4d staining was used as a surrogate for DSA in accordance with the Banff 2022 diagnostic criteria. ABMR and TCMR were classified and subclassified according to the Banff 2022 histopathological criteria.

Clinical variables

The following demographic and clinical variables were collected: recipient age at the time of biopsy, recipient sex, time from kidney transplantation to biopsy, histopathological diagnosis according to the Banff 2022 Classification, estimated glomerular filtration rate (eGFR) 12 months after the index biopsy, and the presence of proteinuria (>0.3 g/24 h) 12 months after the index biopsy. eGFR was calculated using the 2021 CKD-EPI creatinine equation based on recipient age, sex, and serum creatinine concentration. Time from transplantation to biopsy was categorized as early (<3 months), intermediate (3-12 months), or late (>12 months).

Data collection

Clinical and pathological data were retrospectively collected from patients' medical records and the institutional kidney transplant database maintained by the Department of Pathology. Data were extracted using a standardized data collection form developed specifically for this study.

Statistical analysis

Data were entered and analyzed using Microsoft Excel 2016 (Microsoft Corporation, Redmond, WA, USA). Continuous variables were summarized as mean ± standard deviation (SD), whereas categorical variables were expressed as frequencies and percentages. Histopathological diagnoses, classified according to the Banff 2022 Classification, were descriptively evaluated according to the time from kidney transplantation to biopsy and graft function 12 months after the index biopsy, assessed by eGFR and the presence of proteinuria. Owing to the descriptive nature of the study, no inferential statistical analyses were performed.

Ethical considerations

The study protocol was approved by the Scientific Council and the Institutional Ethics Committee of Hospital Clínico Quirúrgico Hermanos Ameijeiras before the initiation of the study. Given the retrospective observational design, only existing clinical records and archived biopsy specimens were reviewed, and no patient-level interventions were performed. All data were anonymized before analysis to ensure patient confidentiality. The study was conducted in accordance with the ethical principles of the Declaration of Helsinki and the institutional regulations governing research involving human participants.

## Results

The demographic characteristics of the recipients are summarized in Table 2. A total of 119 kidney transplant recipients were included in the study. Among the recipients, 72 (60.5%) were male, and 47 (39.5%) were female. At the time of biopsy, most recipients were 36-60 years of age, accounting for 71 (59.7%) biopsies, followed by those aged 19-35 years, accounting for 44 (37.0%), whereas only four (3.3%) recipients were older than 60 years.

**Table 2:** Recipient demographic characteristics

Variable	Category	n (%)
Sex	Female	47 (39.5)
	Male	72 (60.5)
Recipient age at biopsy (years)	19-35	44 (37.0)
	36-60	71 (59.7)
	>60	4 (3.3)

The distribution of kidney allograft biopsies according to the Banff 2022 Classification is summarized in Table 3. Other causes of allograft injury represented the most frequent diagnostic category, accounting for 52 (43.7%) biopsies, followed by TCMR, identified in 37 (31.1%) biopsies.

**Table 3:** Distribution of kidney allograft biopsies according to the Banff 2022 classification ABMR: antibody-mediated rejection; MVI: microvascular inflammation/injury; TCMR: T cell-mediated rejection; IFTA: interstitial fibrosis and tubular atrophy

Banff category	Diagnosis	n (%)
Category 1	Normal biopsy or nonspecific changes	11 (9.2)
Category 2	ABMR/MVI	14 (11.8)
Category 3	Suspicious (borderline) for acute TCMR	2 (1.7)
Category 4	TCMR	37 (31.1)
Category 5	IFTA	3 (2.5)
Category 6	Other changes not considered to be caused by rejection	52 (43.7)

The distribution of histopathological diagnoses according to the time from kidney transplantation to biopsy is summarized in Table 4. During the early post-transplant period (<3 months), 78 (65.5%) biopsies were performed. Acute cyclosporine nephrotoxicity was the most frequent diagnosis, accounting for 15 (19.2%) biopsies, followed by ATN and acute TCMR grade IA, each identified in 12 (15.4%) biopsies.

**Table 4:** Distribution of histopathological diagnoses according to the time from kidney transplantation to biopsy ABMR: antibody-mediated rejection; TCMR: T cell-mediated rejection; IFTA: interstitial fibrosis and tubular atrophy; ATN: acute tubular necrosis; PTLD: post-transplant lymphoproliferative disorder; FSGS: focal segmental glomerulosclerosis; MN: membranous nephropathy; caABMR: chronic active antibody-mediated rejection

Histopathological diagnosis	<3 months, n=78 (65.5%)	3-12 months, n=15 (12.6%)	>12 months, n=26 (21.9%)	Total, n=119 (100%)
Category 1: normal biopsy or nonspecific changes	11 (14.1)	-	-	11 (9.2)
Category 2: ABMR				
Active ABMR	6 (7.7)	2 (13.3)	1 (3.8)	9 (7.6)
caABMR	-	1 (6.7)	4 (15.4)	5 (4.2)
Category 3: suspicious (borderline) for acute TCMR	2 (2.6)	-	-	2 (1.7)
Category 4: TCMR				
Acute TCMR IA	12 (15.4)	2 (13.3)	1 (3.8)	15 (12.6)
Acute TCMR IB	10 (12.8)	-	2 (7.7)	12 (10.1)
Acute TCMR IIA	1 (1.3)	-	-	1 (0.8)
Acute TCMR IIB	3 (3.8)	-	-	3 (2.5)
Chronic active TCMR grade IA	-	-	4 (15.4)	4 (3.4)
Chronic active TCMR grade IB	-	-	2 (7.7)	2 (1.7)
Category 5 IFTA				
Grade I (mild)	-	-	3 (11.5)	3 (2.5)
Category 6: other changes not considered to be caused by rejection				
ATN	12 (15.4)	-	-	12 (10.1)
Acute cyclosporine nephrotoxicity	15 (19.2)	8 (53.3)	-	23 (19.3)
ATN+acute cyclosporine nephrotoxicity	2 (2.6)	1 (6.7)	-	3 (2.5)
Tubulointerstitial nephritis of possible viral etiology	1 (1.3)	-	2 (7.7)	3 (2.5)
Recurrent MN	1 (1.3)	1 (6.7)	-	2 (1.7)
Recurrent FSGS	2 (2.6)	-	-	2 (1.7)
PTLD	-	-	3 (11.5)	3 (2.5)
Chronic cyclosporine nephrotoxicity	-	-	4 (15.4)	4 (3.4)

Between three and 12 months after transplantation, 15 (12.6%) biopsies were performed. Acute cyclosporine nephrotoxicity remained the most frequent diagnosis, accounting for eight (53.3%) biopsies, followed by active ABMR and acute TCMR grade IA, each identified in two (13.3%) biopsies.

Beyond the first post-transplant year (>12 months), 26 (21.9%) biopsies were performed. Chronic active TCMR was the most frequent diagnosis, accounting for six (23.1%) biopsies, including grades IA and IB, followed by chronic active antibody-mediated rejection (caABMR) and chronic cyclosporine nephrotoxicity, each identified in four (15.4%) biopsies.

Graft function 12 months after the index biopsy according to histopathological diagnosis is summarized in Table 5. Patients with immune-mediated lesions generally exhibited mean eGFR values below 40 mL/min/1.73 m², except for those with acute TCMR grade IA.

**Table 5:** Graft function 12 months after the index biopsy according to histopathological diagnosis ABMR: antibody-mediated rejection; TCMR: T cell-mediated rejection; IFTA: interstitial fibrosis and tubular atrophy; ATN: acute tubular necrosis; PTLD: post-transplant lymphoproliferative disorder; FSGS: focal segmental glomerulosclerosis; MN: membranous nephropathy; SD: standard deviation; eGFR: estimated glomerular filtration rate; caABMR: chronic active antibody-mediated rejection

Histopathological diagnosis	n	eGFR (mL/min/1.73 m²), mean±SD	Proteinuria (>0.3 g/24 h), n (%)
Category 1: normal biopsy or nonspecific changes	11	43.0±12.9	0 (0%)
Category 2: ABMR			
Active ABMR	9	29.6±4.1	2 (22.2)
caABMR	5	37.6±21.3	4 (80.0)
Category 3: suspicious (borderline) for acute TCMR	2	30±4.2	0 (0%)
Category 4: TCMR			
Acute TCMR IA	15	48.2±28.3	4 (26.7)
Acute TCMR IB	12	21.9±3.6	7 (58.3)
Acute TCMR IIA	1	17.0	1 (100)
Acute TCMR IIB	3	24±3	3 (100)
Chronic active TCMR grade IA	4	25.5±6.0	3 (75.0)
Chronic active TCMR grade IB	2	23±2.8	0 (0%)
Category 5: IFTA	3	16.6±4.0	2 (66.7)
Category 6: other changes not considered to be caused by rejection			
ATN	12	48.3±30.2	1 (8.3)
Acute cyclosporine nephrotoxicity	23	45.1±22.7	2 (8.7)
ATN+acute cyclosporine nephrotoxicity	3	33.6±9.0	1 (33.3)
Tubulointerstitial nephritis of possible viral etiology	3	39±8.6	1 (33.3)
Recurrent MN	2	32±4.2	2 (100)
PTLD	3	24.6±3.2	2 (66.7)
Chronic cyclosporine nephrotoxicity	4	32±6.7	1 (25.0)
Recurrent FSGS	2	25.5±4.9	2 (100)

Patients with normal biopsies and those with non-immunological lesions, including acute tubular necrosis (ATN) and acute cyclosporine nephrotoxicity, generally had mean eGFR values above 40 mL/min/1.73 m² and a lower frequency of proteinuria at 12 months after the index biopsy. All patients with recurrent glomerular diseases, including recurrent membranous nephropathy (MN) and recurrent focal segmental glomerulosclerosis (FSGS), had proteinuria at 12 months after the index biopsy. Patients with IFTA exhibited the lowest mean eGFR (16.6±4.0 mL/min/1.73 m²), with proteinuria present in two (66.7%) of three patients.

## Discussion

In this retrospective study, kidney allograft biopsies were re-evaluated according to the Banff 2022 Classification to describe their clinicopathological distribution and graft function 12 months after the index biopsy. Non-rejection causes of allograft injury represented the largest diagnostic group, followed by TCMR and ABMR. Histopathological diagnoses exhibited distinct temporal patterns after transplantation. During the first three months, calcineurin inhibitor (CNI) nephrotoxicity was the most frequent diagnosis, followed by ATN and acute TCMR grade IA, whereas after the first post-transplant year, chronic active TCMR was the most frequent diagnosis, followed by caABMR and chronic cyclosporine nephrotoxicity. At 12 months after the index biopsy, immune-mediated lesions generally showed lower mean eGFR values and more frequent proteinuria, except in patients with acute TCMR grade IA. In contrast, normal or nonspecific findings and non-immunological lesions, particularly ATN and acute cyclosporine nephrotoxicity, showed comparatively higher mean eGFR values and a lower frequency of proteinuria.

The demographic characteristics of our cohort were generally consistent with those reported in previous kidney transplant biopsy series. Male recipients accounted for 72 (60.5%) of the study population, and most recipients were younger than 60 years. A male predominance among kidney transplant recipients has also been reported in other retrospective biopsy cohorts, including those reported by Giri et al., with 168 (73.9%) male recipients, and Munib et al., with 51 (92.73%) male recipients [7,8].

Most recipients in our cohort were between 36 and 60 years of age, accounting for 71 (59.7%) of the study population. This finding differs from that reported by Sleem et al., in whose study the largest proportion of transplant recipients belonged to the 16-30-year age group, accounting for 78 (39%) of their cohort. Overall, these findings demonstrate differences in recipient age distribution across transplant cohorts [9].

The distribution of histopathological diagnoses in our cohort reflects the broad spectrum of pathological processes responsible for kidney allograft dysfunction. Other causes of allograft injury represented the largest diagnostic group, accounting for 52 (43.7%) biopsies, followed by TCMR in 37 (31.1%) biopsies and ABMR in 14 (11.8%) biopsies. A similar predominance of non-rejection lesions has been reported by Munib et al., in whose study Banff category 6 accounted for 19 (36.5%) of biopsies, with CNI nephrotoxicity representing the most frequent diagnosis within this category, accounting for 17 (89.4%) cases [8]. In contrast, Saleem et al. found that the miscellaneous (Banff category 5) group was the most common histopathological category, comprising 100 (50%) of biopsies, whereas Yıldırım et al. reported caABMR as the predominant diagnosis, accounting for 14 (31.8%) cases [9,10].

During the first three months after transplantation, CNI nephrotoxicity was the most frequent histopathological diagnosis, accounting for 15 (19.2%) biopsies, followed by acute TCMR grade IA and ATN, each accounting for 12 (15.4%). CNIs remain a cornerstone of maintenance immunosuppressive therapy after kidney transplantation; however, their narrow therapeutic window is associated with a risk of nephrotoxicity. Cyclosporine-associated nephrotoxicity was the most frequent finding in our cohort during this early post-transplant period. However, immunosuppressive regimens and drug levels were not systematically collected, limiting further interpretation of this finding.

Histopathological evaluation is particularly valuable in this setting because the clinical manifestations of CNI toxicity are nonspecific and drug concentrations do not always correlate with the severity of tissue injury, making kidney biopsy important for distinguishing drug toxicity from other causes of allograft dysfunction. Different patterns have been reported in other cohorts. Setoguchi et al. reported active ABMR in six (8%) patients at three months after transplantation [11]. Similarly, Parajuli et al., evaluating causes of early graft failure, reported acute rejection in 102 (31%) cases, including 23 (23%) attributed to ABMR or mixed rejection and eight (8%) to acute cellular rejection [12].

Although ATN is traditionally considered a principal histopathological correlate of delayed graft function (DGF) in the early post-transplant period, its predominance was not observed in our cohort. This differs from Castro Filho et al., who identified ATN as the predominant histopathological diagnosis, accounting for 150 (42.1%) biopsies, followed by acute rejection in 96 (26.9%), and from Lechevallier et al., who reported ATN as the cause of delayed graft function in 70 (92.1%) patients, compared with acute rejection in six (7.9%) [13,14]. Differences in patient populations, biopsy indications, immunosuppressive protocols, and clinical practices across transplant centers may contribute to the variation in histopathological findings reported during the early post-transplant period.

Graft function 12 months after the index biopsy varied across histopathological diagnostic categories. Immune-mediated lesions generally showed mean eGFR values below 40 mL/min/1.73 m² and more frequent proteinuria, with acute TCMR grade IA showing comparatively better graft function. In contrast, patients with normal biopsies and those with non-immunological lesions, particularly ATN and acute cyclosporine nephrotoxicity, showed comparatively higher mean eGFR values and less frequent proteinuria.

Similar findings regarding graft function have been reported by Gunduz et al. In their study, cellular and/or antibody-mediated acute rejection was the most common pathological diagnosis, and higher serum creatinine and urine protein-to-creatinine ratio at the time of biopsy were associated with an increased risk of allograft loss. They also reported that patients with both cellular and ABMR had worse allograft survival than those with either type of rejection alone [15].

All kidney allograft biopsies were retrospectively re-evaluated by an experienced renal pathologist according to the Banff 2022 Classification, providing a standardized histopathological assessment. Graft function 12 months after the index biopsy was assessed using eGFR and proteinuria. However, this retrospective, single-center study had a relatively small sample size, and its descriptive design without inferential statistical analyses precluded assessment of independent associations or prognostic value. DSA testing, molecular diagnostics, immunohistochemical testing for viral pathogens, including SV40 and CMV, and systematic data on immunosuppressive regimens and CNI levels were unavailable. C4d immunohistochemistry was available for ABMR assessment. Future multicenter studies with larger cohorts and prospective follow-up are warranted.

The findings of this study highlight the importance of integrating histopathological findings with clinical parameters, particularly eGFR and proteinuria, for a comprehensive interpretation of kidney allograft biopsies. The Banff 2022 Classification provides a standardized framework for the diagnosis and reporting of kidney allograft pathology, facilitating consistent interpretation and communication between pathologists and transplant clinicians. Continued application of updated Banff criteria may contribute to a better characterization of allograft pathology in relation to clinical graft function.

## Conclusions

Kidney allograft biopsies re-evaluated according to the Banff 2022 Classification demonstrated a broad spectrum of histopathological diagnoses, with non-rejection causes of allograft injury representing the most frequent findings. Histopathological diagnoses showed distinct temporal patterns according to the time from transplantation to biopsy, with CNI nephrotoxicity predominating during the first three months after transplantation. At 12 months after the index biopsy, immune-mediated lesions generally showed lower eGFR and more frequent proteinuria, whereas normal biopsies and non-immunological lesions, particularly ATN and acute cyclosporine nephrotoxicity, showed comparatively better graft function.
